# Canine melanoma: A review of diagnostics and comparative mechanisms of disease and immunotolerance in the era of the immunotherapies

**DOI:** 10.3389/fvets.2022.1046636

**Published:** 2023-01-06

**Authors:** Valentina B. Stevenson, Shawna Klahn, Tanya LeRoith, William R. Huckle

**Affiliations:** ^1^Department of Biomedical Sciences and Pathobiology, Virginia-Maryland College of Veterinary Medicine, Virginia Tech, Blacksburg, VA, United States; ^2^Small Animal Clinical Sciences, Virginia-Maryland College of Veterinary Medicine, Virginia Tech, Blacksburg, VA, United States

**Keywords:** PD-1, PD-L1, PD-L2, dog melanoma, tumor infiltrating lymphocytes, immunotherapy

## Abstract

Melanomas in humans and dogs are highly malignant and resistant to therapy. Since the first development of immunotherapies, interest in how the immune system interacts within the tumor microenvironment and plays a role in tumor development, progression, or remission has increased. Of major importance are tumor-infiltrating lymphocytes (TILs) where distribution and cell frequencies correlate with survival and therapeutic outcomes. Additionally, efforts have been made to identify subsets of TILs populations that can contribute to a tumor-promoting or tumor-inhibiting environment, such as the case with T regulatory cells versus CD8 T cells. Furthermore, cancerous cells have the capacity to express certain inhibitory checkpoint molecules, including CTLA-4, PD-L1, PD-L2, that can suppress the immune system, a property associated with poor prognosis, a high rate of recurrence, and metastasis. Comparative oncology brings insights to comprehend the mechanisms of tumorigenesis and immunotolerance in humans and dogs, contributing to the development of new therapeutic agents that can modulate the immune response against the tumor. Therapies that target signaling pathways such as mTOR and MEK/ERK that are upregulated in cancer, or immunotherapies with different approaches such as CAR-T cells engineered for specific tumor-associated antigens, DNA vaccines using human tyrosinase or CGSP-4 antigen, anti-PD-1 or -PD-L1 monoclonal antibodies that intercept their binding inhibiting the suppression of the T cells, and lymphokine-activated killer cells are already in development for treating canine tumors. This review provides concise and recent information about diagnosis, comparative mechanisms of tumor development and progression, and the current status of immunotherapies directed toward canine melanoma.

## Introduction

Melanoma is the one of the most aggressive and metastatic types of cancer in humans and dogs (1–4). In humans, melanoma accounts for 80% of skin cancers (1, 2). Early detection is a major factor in observed outcomes: for patients with an early diagnosis, the 5-year survival rate is 90%, while this rate decreases dramatically to 10% in patients diagnosed with advanced melanoma (5), where the median overall survival is less than a year (6). Additionally, melanoma is highly resistant to conventional therapies, and new cases are on the increase every year. Melanoma diagnoses have increased annually worldwide by 2.6% and more than 3% in countries like the US, UK, Sweden, and Norway (7, 8).

Chemotherapy after surgical excision was the standard treatment for many years, but overall survival remained low especially for the latest stages of melanoma (9). In the last decade, the understanding of the mechanisms and pathways leading to melanoma and the discovery of effective immunotherapies have shown great promise as more effective treatments for this neoplasm (3, 4, 10, 11). Mutations in the RAS and RAF family of oncogenes have been detected in a significant percentage (15 and 50%, respectively) of human melanoma patients (1, 12), leading to the development of BRAF and MEK inhibitors as potential therapeutic agents (5). Likewise, great progress has been made in the understanding of how the immune system communicates with tumor cells in melanoma and conversely how cancerous cells can modulate the immune response of the patient, establishing the rationale for the development of therapeutic monoclonal antibodies against immune checkpoint molecules (6). Currently, two classes of checkpoint molecules inhibitors have been approved by the FDA, monoclonal antibodies against Programed Cell Death Protein-1 (PD-1), PD-Ligand 1 (PD-L1), and against Cytotoxic T Lymphocyte Antigen-4 (CTLA-4) (13), and more are under development, including anti-PD-L2 monoclonal antibodies (14).

Similarly, canine melanoma is a very aggressive neoplasm, accounting for 3–8% of all neoplastic diseases in dogs and a median overall survival of 8–12 months after diagnosis (15, 16). The most common location for canine melanoma is the oral cavity, accounting for up to 35.8% of all malignant tumors at that site (3), with occurrence at lower frequencies in the skin, eye, and digits (15). In contrast to the human disease, virtually no driver mutations have been identified for canine melanomas (17–19), although investigators are continuing to probe the MAPK signaling pathway since key proteins in this cascade are up-regulated in melanoma (20). As yet, no effective treatments for canine melanoma have been developed that parallel the recent advances made in treating the human disease.

Considering that melanoma is one the most aggressive cancers in both humans and dogs, it is crucial to devise new treatments, especially for advanced cases where available options are of limited efficacy (21, 22). After three decades using conventional modalities, the discovery of immunotherapies is transforming how this disease can be managed (22). In this review, we will address important pathways in the pathogenesis of melanoma, along with the fundamentals of diagnosis, new immunotherapies that have been developed, and their efficacy for the treatment of malignant melanoma in humans. We endeavor to highlight the promise, as well as the pitfalls, of translating mechanistic insights and thus therapeutic opportunities between humans and dogs.

## Morphology and features of canine melanoma

### Origin and histologic features

Melanomas arise from melanocytes, which have an embryologic origin in melanoblasts that are derived from the neuroectoderm of the neural crest (23). Melanoblasts migrate from the neural crest to the integumentary system and localize within the epidermis and hair follicles where they differentiate into melanocytes or remain as melanocyte stem cells (23, 24). Melanin production by melanocytes occurs in cytoplasmic organelles called melanosomes, *via* a reaction catalyzed by tyrosinase through a chain of conversions that transform tyrosine to DOPA and on to melanin. The resulting granules of melanin are then transferred from melanocytes to keratinocytes by a membrane vesicle-mediated process (25, 26). This is the usual process for pigmentation of dermal keratinocytes by melanocytes. An alternative pathway provides for production of melanocytes from a bipotential precursor cell (Schwann/melanoblast) that migrates along nerve sheaths (24, 27). This process is regulated in part by microphthalmia-associated transcription factor (MITF) and the receptor tyrosine kinase KIT and its ligand (15, 27, 28).

**Figure 1:**
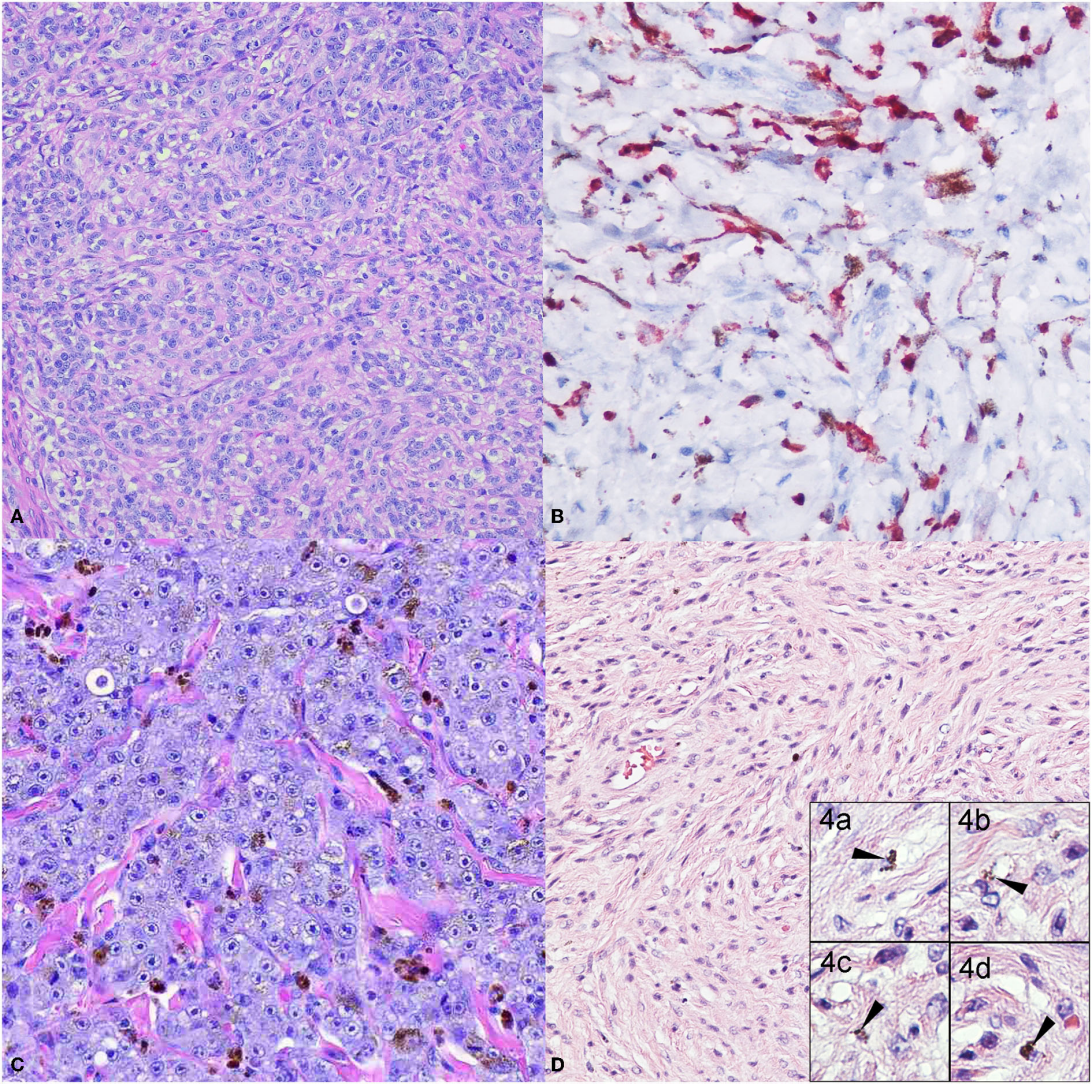
Common canine melanoma morphologies. The panel shows the more classical melanoma morphologies. **(A)** Dog oral melanoma, 20X. Amelanotic, cords of polygonal cells with no apparent intracytoplasmic melanin granules. Neoplastic cells have a large nucleus with a prominent nucleolus. **(B)** Dog oral melanoma 20X. Amelanotic melanoma immunolabeled for Melan-A detected in the cytoplasm. (**C)** Dog oral melanoma 20X. Epithelioid melanoma with polygonal cells arranged in cords and nests, with scattered intracytoplasmic melanin granules. **(D)** Dog cutaneous melanoma 20X. Fusiform melanoma, elongated to spindloid cells arranged in streaming bundles, with occasional intracytoplasmic melanin granules (inset 4.a-d, 40X).

The diagnosis of melanomas can be challenging, as they can resemble other types of tumors such as carcinomas, sarcomas, lymphomas, or tumors of an osteogenic origin (29). In addition, even though most melanomas have very characteristic melanin granules in their cytoplasm, they can also be amelanotic (Figures 1A, B). In these instances, the recognition of morphologic features described by Smedley et al. and the use of immunohistochemical (IHC) markers are very useful tools (30, 31). In addition to their intracytoplasmic melanin granules, melanomas can feature varied intratumor cell morphology, the presence of neoplastic cells at the epidermal-dermal or mucosal-submucosal junction, finely stippled to vesiculated nuclei, and often a single central and prominent nucleolus (31). Several histological morphologies have been described for melanoma: epithelioid (Figure 1C) with polygonal melanocytes arranged in cords or nests is the most frequently diagnosed; next, fusiform (Figure 1D) with spindloid cells arranged in streaming bundles; mixed with a combination of both polygonal and spindloid morphologies; small-round with cells arranged in cords; and, in cases of more undifferentiated tumors, melanomas can exhibit a neuron-like appearance (15, 31–34). In less frequency, cellular, balloon cell, signet ring, and clear cell morphologies have been reported (31).

### Diagnostic criteria

As melanoma can resemble multiple tumor types, IHC has become a valuable diagnostic tool. Whereas, SOX10, S100, HMB-45 and Melan A are normally used as markers of human melanoma, (35, 36) common markers used to identify canine melanomas are Melan-A, PNL2, and tyrosinase related protein 1 and 2 (TRP-1 and TRP-2) (29, 30). Melan-A (Figure 1B) is a protein marker specific for melanocyte differentiation, and its immunolabeling is highly sensitive and specific (29, 37). PNL2 is a monoclonal antibody that recognizes an as-yet unidentified cytoplasmic melanocytic antigen and is described as exhibiting staining specificity similar to that of anti-Melan-A (29). Tyrosinase, the third most-common marker, is a cytoplasmic protein Involved in melanocyte differentiation and melanin biosynthesis (29). It has been reported that immunolabeling for these markers is reduced in tumors with spindloid or undifferentiated morphology (29). These three markers combined can reach a sensitivity of 100% (29, 30). Other markers such as S100 have been suggested in the past for humans and dogs, with good results in canine melanoma cell lines (38), but its specificity is considered low by other investigators (29, 30), similar to results for vimentin (37). HMB-45 (Human Melanoma Black) has also been reported in canine melanoma with high variability among the few reports available, and its use is not recommended in canine neoplasms (31).

Another antigen, more recently recognized, is chondroitin sulfate proteoglycan-4 (CSPG4), also known as high molecular weight-melanoma associated antigen (HMW-MAA), an early cell-surface progression marker associated with proliferation, migration, and invasion. This maker, first described in human melanoma but lately reported in canine melanoma, has promising potential therapeutic use as the basis for a DNA vaccine (39–42), and has been reported to be expressed more frequently in amelanotic melanomas (40).

**Figure 2:**
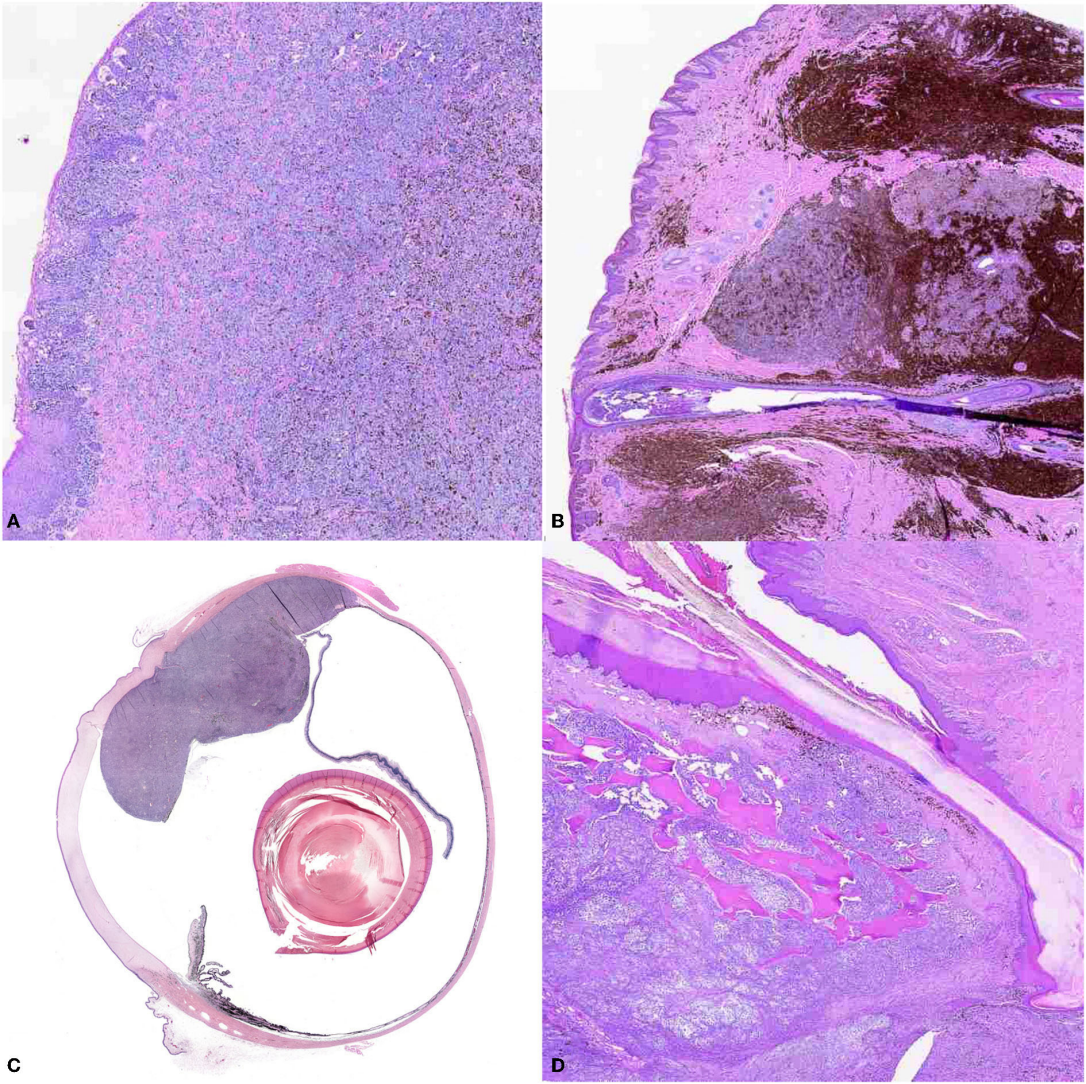
The panel shows the different locations of canine melanoma. **(A)** Oral melanoma (10X). Small nests of neoplastic melanocytes infiltrate the mucosal epithelium, and large sheets of neoplastic cells obscure the propria mucosa. Brown intracytoplasmic granules consistent with melanin are throughout the neoplasm. **(B)** Cutaneous melanoma (10X), haired skin. Large nests of neoplastic melanocytes infiltrate the dermis. Numerous melanomacrophages infiltrate the periphery of the neoplasm. **(C)** Ocular melanoma (montage of scan at 10X). A large, multilobulated neoplasm extends from and occupies the back of the eye. **(D)** Digital melanoma (10X). Sheets of neoplastic melanocytes obscure the dermis and infiltrate the bone.

Although originating most frequently at oral, cutaneous, digital, subungual, or ocular sites (Figure 2), melanomas can also occur in the gastrointestinal tract, nervous system, and muco-cutaneous junctions (33). Traditionally, oral melanoma has been most closely associated with malignancy, whereas the cutaneous counterpart typically has a relatively benign course of disease, and digit or subungual with increased rate of recurrence. Ocular melanoma has been described to be locally aggressive, but with limited metastatic potential (15, 18, 31, 33, 34, 43, 44). However, it must be emphasized that location alone should not be used to determine prognosis (44).

The main features used for prognostic determination were nuclear atypia, growth fraction, and mitotic index, as reported by Smedley et al. and supported by the most recent melanoma consensus, revised in 2020 by the Veterinary Cancer Society/American College Veterinary Pathology Oncology-Pathology Working Group (44). Nuclear atypia was found to be highly correlated with outcome, especially for pigmented melanomas with epithelioid morphology. The authors suggest a threshold of >30% of cells with atypical nuclei from a total of 200 cells observed as indictive of a poor prognosis for oral and lip neoplasms and >20% of cells with atypical nuclei for cutaneous neoplasms (31).

Growth fraction (or Ki67 index) is measured by using IHC for the Ki67 protein (Ki67p). Detection of Ki67p is indicative of mitotic activity of intrinsic cell populations, as it is only present in actively proliferating cells (i.e., in G_1_-M phases of the cell cycle but not G_0_) and is commonly used as a marker for aggressiveness of the neoplasms (45). For oral melanomas, Ki67p is determined by calculating the average number of positively labeled melanocytes over a total of five areas of 1 mm^2^ optical grid at 400x magnification. In cutaneous melanomas, the Ki67 index is determined by estimating the percentage of positive labeled melanocytes per 500 counted cells. Ki67p is significantly different for benign and malignant melanocytic neoplasms, with increased numbers of immunolabeled cells found in oral melanomas and associated with a poor prognosis and reduced survival (31),

Regarding the mitotic index, briefly, it is well-established that oral and lip melanocytic neoplasms with >4 mitoses per 2.37 mm^2^ observed microscopic field have an increased risk of death within 1 year of diagnosis, and, for cutaneous and digital melanocytic neoplasms, >3 mitoses in 2.37 mm^2^ are statistically correlated with low 2-year survival rate (31). In addition, the VCS/ACVP consensus group has suggested addition of tumor thickness to the prognosis indicators for cutaneous melanomas (44). A tumor thickness of >0.95 cm is associated with poor prognosis and increased risk of recurrence and developing metastasis (46). The authors also reported that tumor thickness is greater in melanoma than melanocytoma.

## Genetic characterization of melanoma

### Genomic contributors to tumorigenesis in human melanoma

Melanoma is considered one of the cancers with the highest degree of mutations (47, 48), and four major subtypes based on the genetic mutation burden have been described in humans. The first three groups encompass activating driver mutations in B-Raf proto-oncogene serine/threonine kinase (BRAF), the GDP/GTP binding protein RAS (RAS), and the tumor suppressor Neurofibromatosis Factor 1 (NF1), respectively, while the fourth group (“Triple Wild Type”) lacks any of these three alterations, although it may have a low frequency of other mutations such as those observed in KIT (12). The detection of these of mutations in patients informs the therapeutic plan and gives insight into their prognosis. Some patients with mutated BRAF could benefit from targeted therapies such as BRAF and MEK inhibitors, and, while these inhibitors might not be effective for some other patients, they could still benefit from other approaches including immunotherapies (22, 49).

The BRAF mutation is a somatic missense point mutation that results in a single substitution of glutamic acid for valine at amino acid 600 (V600E) in human BRAF, conferring a constitutively active state to the BRAF protein kinase. This translates into a continuous state of survival of the cell, increasing proliferation and promotion of metastasis (50). The BRAF mutation has been described in 40–50% of all human melanoma cases (34, 47, 49) and is more commonly seen in younger patients (12). Additionally, in patient with nevi that have the BRAF mutation, a second hit could induce malignancy by an inactivation mutation in the phosphatase and tensin protein (PTEN), since this protein has a role as a negative regulator of cell growth and survival signaling pathways (47, 51).

Mutation of RAS is the second most common genetic alteration in human melanoma, present in 15–25% of all cases (47, 49). This mutation alters the RAS protein and maintains protein activation, which continuously stimulates the mitogen/extracellular signal-regulated kinase (MEK/ERK) pathway and in some cases can also lead to the activation of the Phosphatidylinositol-3-kinase (PI3K) signaling pathway, driving cell survival and proliferation (49). Mutation in Neurofibromatosis Factor 1 gene (NF1) is the third most common mutation in melanoma and induces loss of the regulatory effect of NF1 on the RAS protein (48, 49). Consequently, RAS is continuously activated, and the cell enters into a proliferative state. This mutation is more frequently detected in older patients and is associated with better response to checkpoint molecule blockade therapies (12) as described below.

The fourth group is the Triple Wild Type, lacking BRAF, RAS and NF1 mutations. Even though melanomas in this group do not have any of the three mutations described above, they can have other mutations like GNAQ (G signaling protein) and c-KIT (CD117, a tyrosine kinase receptor for stem cell growth factor) in a lower frequency (< 7%); these proteins are associated with intracellular signaling and can result in uncontrolled cell proliferation (12, 52). Triple Wild Type melanomas are mostly from non-cutaneous origin, such as mucosal melanoma and, while less common, are very aggressive and associated with a poor prognosis (34, 48, 53). Other mutations found in human mucosal melanomas include activating mutations in SF3B1, loss of CDKN2A, PTEN, and SPRED1 (54). PTEN mutation has been reported in canine mucosal melanoma, although its role in disease severity or progression has not been established (55).

Activation of the signaling pathway mTOR (mammalian target of rapamycin), a serine/threonine kinase, has been strongly associated with the development of different neoplasms, including melanoma (34, 56–58). This complex is activated by the PI3K/AKT pathway and regulates cell growth. Its overexpression can lead to aberrant proliferation and increased migration, conferring on the neoplastic cells more invasive capabilities (59–62). There are two main components in this complex: mTORC1, which regulates numerous processes of protein synthesis that promote cell growth and can also inhibit certain catabolic process such autophagy contributing to the malignancy of the neoplasm (60, 63, 64); and mTORC2, associated with control of cellular structure, regulation of the cytoskeleton, and cell survival (60, 61, 63).

Another factor that has been reported commonly in cancer in multiple species is the variability of Somatically-acquired Copy Number Alterations (SCNAs), which consist of deletion or amplification of DNA fragments encoding regulators of cell proliferation (65). Recent studies report similar increases in SCNAs in human and canine mucosal melanoma (55, 66). In canine mucosal melanoma, increased aberrations by gain of chromosome 13 and 17, and loss of chromosome 2 and 22 were reported using comparative genomic hybridization *in situ*, and copy number assessments using fluorescence *in situ* hybridization revealed gain of c-MYC and loss of CDKN2A, whereas in humans there was gain of 1q, 6p, 8q, 7, and loss of 6q and 10 (66, 67).

### Lessons for canine melanoma?

The comparative oncology approach of identifying a mutation burden in canine melanoma similar to that in human disease has met with only limited success, as canine melanomas do not appear to have any of the mutations described above in high frequency. Although the BRAF mutation was found in 3 out of 54 canine melanomas studied (68), it is far less common than reported in the human disease, where it occurs in 40–50% of cases (17–19, 69). Additionally, NRAS and NF-1 mutations in canine melanoma have also been reported, but at a very low frequency (70). Although no predominant mutation has been described for canine melanoma to date, the search continues for a driver mutation that could be involved in the development of melanoma in dogs (20), while other investigators have proposed the use of the dog as an animal model to study Triple Wild Type and mucosal melanoma in humans (18, 32, 34). This parallel approach could bring new perspectives regarding risk factors and experimental paradigms from which both species will benefit. For instance, activation of the mTOR signaling pathway has been also explored in canine melanoma as well as in the human disease, and studies in canine melanoma cell lines have demonstrated activation of this pathway along with the inhibitory efficacy of Rapamycin and other drugs (71–73). Clearly there is much to be learned about the genetic drivers of melanoma in dogs.

## Tumor infiltrating lymphocytes and immune checkpoint molecules—A new frontier for canine melanoma therapy

### Tumor/lymphocyte interactions in the human melanoma microenvironment

Melanoma is considered an immunogenic tumor, and in most of the cases the tumors have a high degree of lymphocytic infiltration. However, most melanomas continue to grow, suggesting that tumor infiltrating immune cells fail to control and modulate tumor invasion (16, 74, 75). Furthermore, the tumor cells may suppress tumor infiltrating lymphocytes (TILs) and thereby escape immune surveillance. Nevertheless, the presence of TILs is associated with better responses to targeted and immune-directed therapies (76–78). Additionally, it has been demonstrated that the presence of TILs typically correlates with a favorable prognosis in melanoma (79–84) and other human cancers (85–88).

In view of this association, a system to characterize TILs infiltration in human melanoma was established by Clark (83) and has been adapted by other investigators for application to canine melanoma and other neoplasias. Briefly, the TIL distribution is classified as absent, brisk, or non-brisk. The “absent” category is described as no lymphocytes directly opposed to the tumor cells; the “non-brisk” category as isolated, focal and/or segmental infiltration in the tumor; and the “brisk” category as TILs segmentally infiltrating either at the entire base of the tumor or diffusely infiltrating within the tumor (83). Focal TILs that are in areas of fibrosis or remain in perivascular spaces but do not penetrate the tumor are also considered under the absent sub-classification (81). Other studies propose a combined system that considers the distribution and density of these TILs (89). These authors also reported that dense TIL infiltration with a brisk distribution was strongly associated with better prognosis in contrast to the non-brisk and absent distribution (83).

TILs comprise a heterogenous population of different lymphocytes bearing the co-receptors for the T cell receptor CD4 or CD8, including CD4 T regulatory cells (Tregs), CD4 T helper cells, CD4 memory cells, and CD8 effector cells (90). A majority of studies that aim to classify the subset of TILs in the tumor microenvironment use IHC markers for surface proteins, such as CD20 and CD79a for B cells, CD3 for T cells, CD4 and CD8 for T cells, and FOXP3 for regulatory T cells (91, 92).

Human melanoma patients with increased TIL abundance in the primary tumor have improved survival times compared to those with low TIL infiltration (79), better progression-free survival (84), and longer disease-free survival (89), while low TIL infiltration is associated with tumor recurrence and distant metastasis (87, 89). However, other investigators suggest that TIL infiltration alone is not predictive of outcome for non-small cell lung cancer and suggest that the subset of these TILs is more important (86), as also described in patients with metastatic colorectal cancers (87). These authors emphasize the importance of characterizing TILs and report that high numbers of CD8 T cells expressing cytolytic enzymes and CD4 T cells lacking inhibitory receptors were associated with a good prognosis when compared with TILs expressing PD-1 (86). Interestingly, the association between high Ki67 expression levels and high TILs levels has been described in triple-negative breast cancer, suggesting that the antitumor immune response is increased in aggressive tumors (93). Based on the findings described above, profiling of TILs in the tumor microenvironment provides a useful framework for understanding the contribution of different subsets of lymphocytes to formation of a tumor-promoting or tumor-inhibiting environment, associated with CD8 T cells or T regulatory cells, respectively.

### Extension of tumor/lymphocyte insights to canine melanoma

Several studies in canine tumors have reported a relationship similar to that described above in human melanoma, associating density of TILs positive for the T cell co-receptor CD3 with favorable outcome in histiocytic sarcoma following resection (94). Another study in canine gliomas determined the nature of the infiltration, differentiating between CD3 T cells and those positive for the Forkhead box protein P3 (FOXP3), a regulator of Treg cell gene expression, finding similar results with generally low numbers of Treg cells, although higher FOXP3 densities were found in high grade tumors (95). Higher levels of Treg cells in peripheral blood and tumor-draining lymph nodes have been reported in dogs with melanomas when compared to healthy controls (96), and has been linked to an increased risk of death (97, 98). As explained in another study, subsets of TILs have an important role on tumor progression and outcomes, as CD8 T cells are key determinants of antitumor immunity, and CD4 T cells can either support this immune response or suppress it, as the case for Treg cells (99). These authors evaluated TILs in canine oral melanoma, taking into consideration both TIL density and distribution using the classification proposed by Clark et al. (described above), reporting that higher CD4 and CD8 TIL levels were found in less aggressive stages and also among primary tumors when compared to recurrent tumors. Additionally, patients with a brisk and non-brisk CD8 T cell infiltration had higher overall survival rates when compared to those with absent infiltration, and no impact on survival was appreciated from variable T reg and CD4 T cell infiltration (99).

Thus, the relationship between TILs and cancerous cells in the tumor microenvironment is an important consideration for the development of therapies targeting the regulatory molecules of the immune system. Tumor infiltration is not limited to tumor growth suppression, since it can also be associated with tumor growth and immunosuppression (100). Importantly, it has been reported that TILs are associated with better responses to targeted and immunotherapies in humans (76–78), especially for melanoma (101).

### Immune checkpoint molecules: The molecular basis of immunosuppressive tumor/lymphocyte interactions

In patients with cancer, lymphocytes can experience chronic exhaustion and express PD-1 and CTLA-4 co-inhibitory receptors. At the same time, cancerous cells can overexpress PD-1 ligand (PD-L1) and CTLA-4 and thus inhibit an antitumor response by the immune system. PD-L1, PD-L2 and CTLA-4 are highly expressed across different cancer types (102, 103). Cancer cells can also recruit immune-suppressor cells like T regulatory lymphocytes, myeloid derived suppressor cells (MDSC), and type 2 macrophages, releasing immunosuppressive cytokines like IL-10, thereby allowing for tumor growth and proliferation, as has been described in several studies (100, 104–106).

Interestingly, expression of PD-1's alternative ligand PD-L2 has been detected in several human tumors, and some studies have reported an overexpression of PD-L2 in absence of PD-L1 (14). Other studies have found PD-L2 to be highly correlated with PD-1 and PD-L1 expression (103, 105) or with cancer infiltration and metastasis (105, 107). The degree to which tumor-expressed PD-L2 can substitute for PD-L1 as an immunosuppressing agent is not well-established, but an association between resistance to anti-PD-L1 immunotherapies and PD-L2 overexpression suggests an important role for PD-L2 in immune escape (108).

After decades of conventional treatment for advanced melanoma with limited success, surgical excision, chemotherapy and radiation are now being complemented with immunotherapies (109). After the discovery of the roles of checkpoint molecules such as PD-1, its ligand PD-L1, and CTLA-4, efforts have been redirected toward developing antibodies to inhibit their activity and release the patient's own immune system to fight cancer (22, 109).

### Current targeted and immunotherapies for melanoma in humans

In humans, patients with BRAF mutations are candidates for targeted therapies such as BRAF and MEK inhibitors. These therapies have been shown to inhibit tumor growth by decreasing the kinase activity in the MEK/ERK signaling pathway (49, 110, 111). Clinical trials have demonstrated that combined therapies are more effective than MEK or BRAF inhibition alone, with higher response rate and longer progression free survival (49). Vemurafenid and Dabrafenid or Trametinib, BRAF-mutant inhibitors approved by the FDA for the treatment of BRAF-V600-mutated-melanoma, have reported remission in 90% of patients (22). However, the clinical benefit is limited by the development of therapy resistance due to mRNA splice variants of BRAF and re-activation of alternate signaling pathways (22, 112, 113). There are several clinical trials using MEK and BRAF inhibitors in combination with immunotherapies that show improving response rates and duration of results, especially for patients unresponsive to BRAF/MEK inhibitor therapy; response rates up to 70% are reported (111, 112, 114).

Another strategy studied for the treatment of melanoma is the activation of anti-cancer immunity, by either stimulating dendritic cells or amplifying T cell activation. For several years, attempts were made using vaccines containing melanoma antigens without success due to the immunostatic effect of the tumor microenvironment or the poor immunogenicity of the antigens, inducing either high toxicity or insufficient responses (22, 109). Similar results were seen with agonistic antibodies for dendritic cells and cytotoxic lymphocytes (OX40 and CX3CL, respectively) (109). However, studies measuring the expression of OX40 in TILs have detected an upregulation of OX40 on T regulatory lymphocytes (115), and anti-OX40 antibodies have been proposed as adjuvants alongside immunotherapeutics (116).

Nowadays, immunotherapy research in multiple species is focused on the development of checkpoint molecule inhibitors and their efficacy in different types of cancers, and dramatic advances have been made in treatment of human disease. A variety of antibodies inhibiting the suppressive effect that cancer cells have over TILs have been developed during the last decades. Immunotherapies that have shown good promise are mononuclear antibodies against CTLA-4, PD-1 and PD-L1 (22, 117–120). Antibodies against checkpoint molecules approved by the FDA in the treatment of metastatic melanoma are Ipilimumab (anti CTLA-4), Nivolumab and Pembrolizumab (anti PD-1) (22). In addition, an anti PD-L1 antibody (Atezolizumab) has been approved for urothelial carcinoma and non-small cell lung cancer (NSCLC) (121). These drugs have an advantage over therapies that target tumors driven by BRAF, thus making them available for patients with other types of mutations or lacking the most frequent mutations. An advantage of the newly-developed Atezolizumab is that, in addition to binding free PD-L1, it also displaces the ligand from PD-1 and appears to be more effective than other anti-PD-L1 antibody drugs currently in clinical trials (121, 122). Additionally, immunotherapies targeting PD-1 and/or PD-L1 have shown fewer side effects than those targeting CTLA-4 (123). A meta-analysis of 19,217 human patients on immune checkpoint inhibitors showed toxicity-related death rates < 0.4% for anti-PD-1 and anti-PD-L1, 1.08% for anti CTLA-4 and 1.23% for combined therapies (124).

### Current immunotherapies in dog cancer

**Figure 3:**
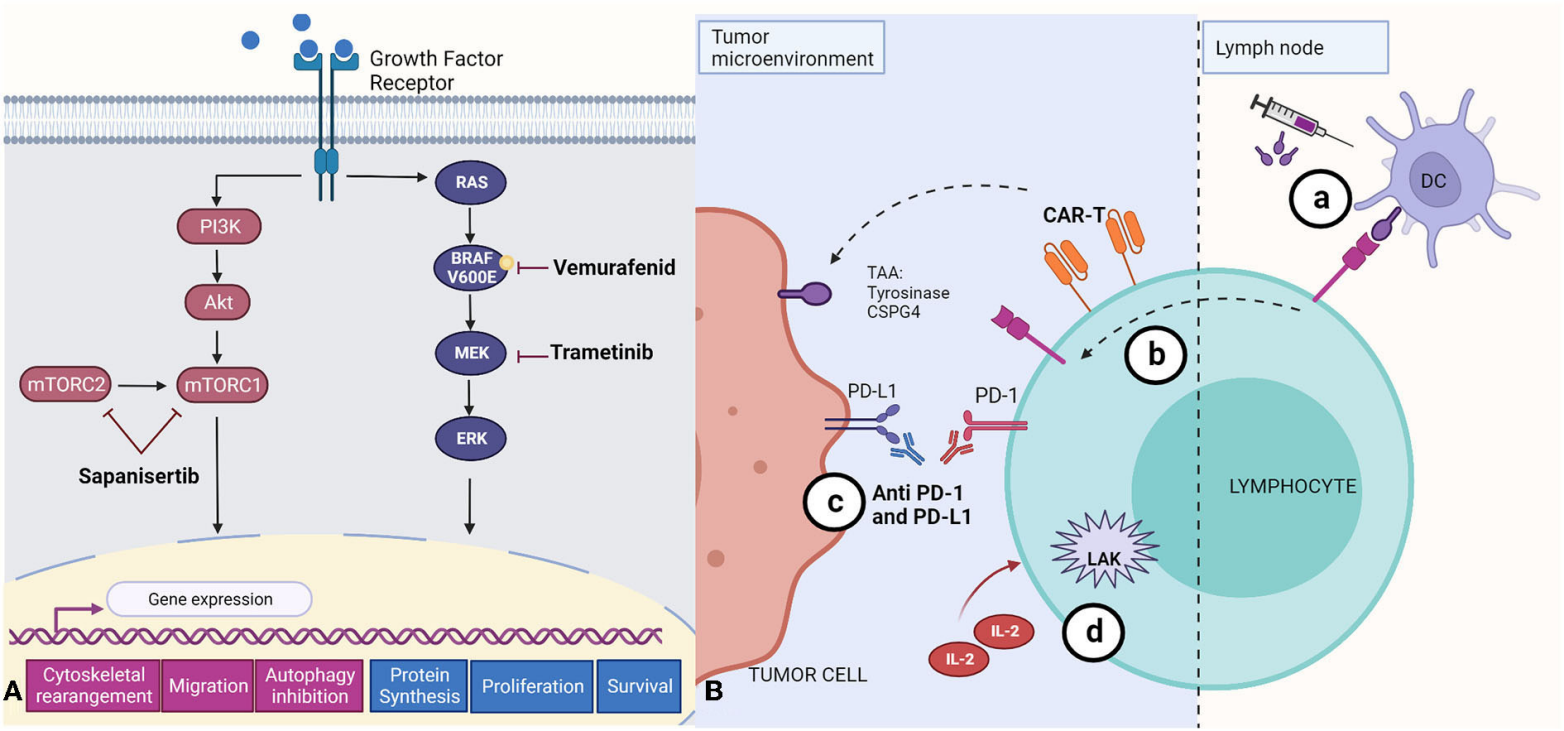
Summary of small molecule- and antibody-based approaches to melanoma treatment. **(A)** Targeted therapies that aim to suppress central mechanisms of cellular proliferation and metastasis by inhibition of the mTOR (Sapanisertib), and MERK/ERK (Vemurafenid, Trametinib) pathways. The mTOR pathway activates gene expression that leads to cytoskeletal rearrangement, migration, and inhibition of autophagy. The RAS/BRAF/MEK/ERK pathway activation leads to increased protein synthesis, and promotes cell proliferation and survival. **(B)** Immunotherapies described to date in canine tumors. (a) Immunogens encoded by DNA vaccines, including tyrosinase (Oncept) or CSPG4, are first recognized by dendritic cells (DC) which later present antigen fragments to T cells at the lymph node; these T cell are able to target the antigen at the tumor microenvironment; (b) CAR-T cells engineered for specific tumor-associated antigens (TAAs) and cloned *in vitro* before being reincorporated into the patient; (c) Monoclonal antibodies recognizing PD-L1 blocking its binding with PD-1 receptor; and (d) Lymphokine-activated killer cells activated *in vitro* with IL-2. Created with BioRender.com.

Exploration of the utility of immunotherapies in canine cancer therapy is in its very early days, with much of the published work having been conducted *in vitro*. For example, combined treatment with trametinib and sapanisertib in order to inhibit both MEK and mTOR pathways (Figure 3A) has been reported to induce apoptosis and reduce cell survival in canine melanoma cell lines (72), and one study reported that inhibition of the mTOR pathway reduced invasion and angiogenesis in hemangiosarcoma cell lines (125). However, combining these drugs with immunotherapies or targeting both pathways resulted in toxicities in studies done in cell lines and xenografts (72, 73).

Availability of immunotherapeutic agents is limited in the treatment of canine cancers. The less comprehensive characterization and understanding of the immune response to tumors in this species is a major obstacle, due in part to limited reagent availability (11). However, recent studies have explored vaccines, adoptive cell transfer, and anti PD-1 and PD-L1 therapy (21, 126, 127). Currently, Oncept, a xenogenic human tyrosinase DNA vaccine, is the sole vaccine approved by the USDA for the treatment of canine melanoma (Figure 3B) (21, 128, 129). Although one study reported improvements in survival times (130), it has been established that the vaccine alone is not enough to maintain anti-tumor efficacy, since the tumor microenvironment can suppress the immune response, and, once lymphocytes have been activated, they can express immunosuppressive checkpoint molecules (11, 21, 128, 129). Since the discovery of CSPG4 antigen in canine melanomas, efforts have been made to develop a DNA vaccine targeting this tumor associated antigen (Figure 3B), seeking to induce a safe immune response with longer overall disease-free survival when used as an adjuvant with other therapies and surgery (39–42, 131).

Additionally, adoptive cell transfer has been explored for the treatment in canine cancers, using lymphokine-activated killer (LAK) cells and CAR-T cells. These therapies have been evaluated for canine osteosarcoma (*in vitro*), B cell lymphoma, and melanoma (*in vitro* and *in vivo*), (11, 100, 128, 132) resulting in high toxicity for osteosarcoma (100) but modest antitumor activity for B cell lymphoma and melanoma (132, 133). Although LAK therapy demonstrated an immune-enhancing effect, it is not sufficient as monotherapy and thus should be evaluated as an adjuvant for other immunotherapies (128, 132). Similarly, CAR-T cells present several disadvantages, such the high cost and time required to engineer the cells, the risk of mutations in the receptor, antigen selection for the engineering of the receptor, and the fact that CAR-T cells are transient and not sustainable over long periods of time (100, 129).

Increased levels of CTLA-4 have also been reported in canine melanocytic tumors and associated with an immune-suppressed tumor microenvironment leading to immune escape and worsened prognosis (98). Great efforts have been made toward the development of a canine anti-CTLA4 monoclonal antibody, with promising results in mouse models (134). Another group has developed a chimeric heavy-chain antibody targeting cells expressing CTLA-4, such as Tregs and CD4 helper T cells, and reported an induction of the cytokine IFN-γ, whose pleiotropic actions include anti-tumor activity (135).

Lastly, one group has made great progress in the detection of overexpression of PD-1 from TILs and PD-L1 from malignant tumors in several canine cancers, such melanoma, osteosarcoma, hemangiosarcoma, and others, in the interest of establishing a rationale for application of anti-PD therapeutics in dogs (136, 137). Anti PD-1 chimeric antibody has been developed by a research group which reported reduction of tumor burden, objective partial response in 26% of the treated dogs with a Stage IV oral malignant melanoma, and a trend of increase of survival times, with maintained complete regression for more than 1 year in 2 cases (127, 138). In a pilot clinical study, these investigators demonstrated that anti-PD-L1 therapy can induce an objective antitumor response for oral melanoma (126). Furthermore, the expression of these checkpoint molecules has also been reported by our laboratory as increased in melanoma when compared with its benign counterpart melanocytoma and is associated with the density of CD3+ TILs (139). Recently, a group tested the cross-reactivity of different FDA-approved human immune checkpoint inhibitors (ICI) in canine tissue, showing two anti PD-L1 (Atezolizumab and Avelumab) with cross-reactivity, and Atezolizumab with the most robust T-cell cytokine production *in vitro* (140). A schematic representation of the immunotherapies in canine cancers is shown in Figure 3B. These studies establish the basis for future research on the development of checkpoint molecule inhibitors as a treatment for melanoma and other cancers in dogs, and it is expected that in the future, these therapies could be an effective and affordable option for the veterinary practice.

## Conclusion

Comparative oncology between humans and dogs will set the foundations for a better understanding of common factors that are associated with the development of melanoma in humans. Additionally, it will provide insight into which factors are associated with the development of melanoma in dogs, and by what mechanism. As dogs spontaneously develop melanoma, are exposed to the same environmental hazards as humans, and have similar physiology to humans, they are excellent candidates for future development of immunotherapies. The evident lack of effective therapies for melanoma in dogs increases the urgency of the search for an effective treatment. Human benefit from immunotherapies has been documented in numerous studies, and it would be remarkable to continue exploring this option for treating dog cancers.

Lastly, study of the frequencies of occurrence, the spatial and temporal distribution, and functional categorization of TILs has great therapeutic potential. As new insights are gathered from recent studies, TILs will provide a representation of patients' immune system and the tumor microenvironment, offering a promising tool for determining therapeutic approaches.

## Author contributions

VS conducted the literature search and drafted and edited the manuscript. SK provided guidance on clinical manifestations of canine melanoma. TL provided guidance on diagnostics. WH provided guidance on molecular mechanisms. SK, TL, and WH contributed to editing of the manuscript. All authors contributed to the article and approved the submitted version.
